# Advances in male sex separation for the support of mosquito control programs

**DOI:** 10.3389/finsc.2026.1773663

**Published:** 2026-03-10

**Authors:** Molly Duman-Scheel

**Affiliations:** 1 Department of Medical and Molecular Genetics, Indiana University School of Medicine, South Bend, IN, United States; 2 Department of Biological Sciences and Eck Institute of Global Health, University of Notre Dame, Notre Dame, IN, United States

**Keywords:** *Aedes*, *Anopheles*, dengue, genetics, IIT, malaria, sex separation, SIT

## Abstract

Several mosquito control technologies, including the sterile insect technique (SIT), the incompatible insect technique (IIT), and a variety of genetic technologies are emerging as promising solutions for combatting insecticide resistance and the spread of vector-borne diseases. These approaches involve mass releases of male mosquitoes in an effort to suppress mosquito populations in an eco-friendly manner. At small scale, male mosquito selection can be achieved through the use of mechanical separation techniques, but such methods are not sufficient for scaled implementation of emerging mosquito population control technologies. This review discusses mechanical, genetic, and automated mosquito sex sorting techniques that have emerged to address the need for scaled male mosquito production, as well as the potential contributions of RNA interference (RNAi) to facilitate this process. One RNAi method utilizes the oral delivery of yeast expressing interfering RNA targeting genes required for female larval survival. The yeast, which can be incorporated into normal insect larval diets, enables male sex selection during larval rearing in mosquitoes and could easily be extended to other insects. RNAi-mediated sex-sorting technologies, in combination with mechanical, genetic, and automated sorting technologies, could facilitate the scaled production of adult males in support of global insect population suppression efforts.

## Graphical Abstract

**Figure:**
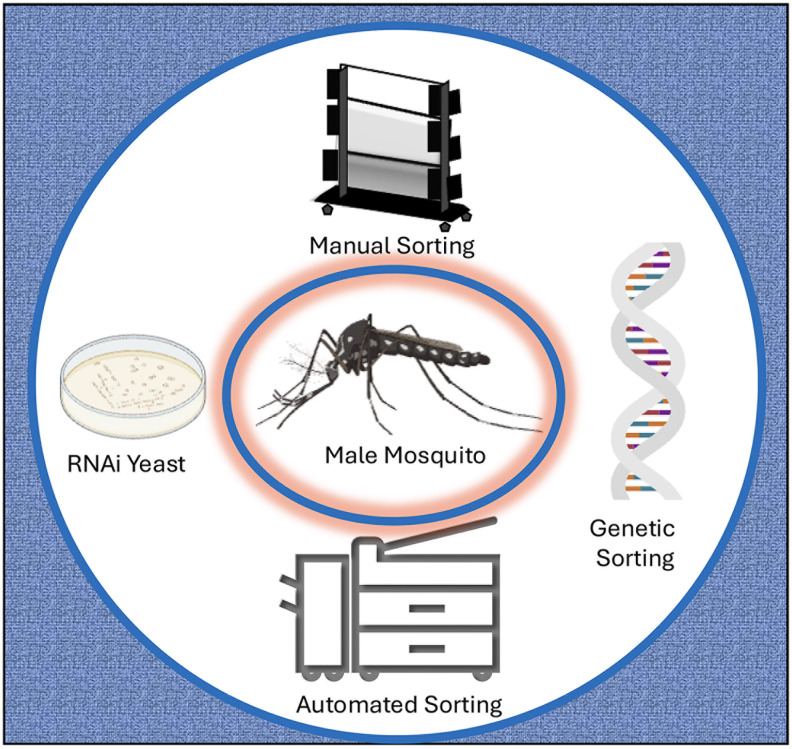
Manual, genetic, automated, and RNAi yeast sorting methods, and combinations thereof, can be used to scale production of male mosquitoes to facilitate new technologies that suppress mosquito populations.

## 1 Introduction

Pathogens spread by mosquitoes are responsible for diseases such as dengue and malaria, which kill ~700,000 people annually (1). In addition to insecticides, population-based control strategies such as the sterile insect technique (SIT) (2–4), the incompatible insect technique (IIT) (5–7), combined SIT and IIT (8, 9), and various genetic technologies that suppress mosquitoes or make them unable to transmit pathogens (10–15) could prove valuable in the fight against mosquito-borne illnesses.

SIT, which involves the release of mating-competitive sterilized males, has been extended to insects of medical importance, including *Aedes* spp. mosquitoes (3) that vector arboviruses such as dengue (1). Sterile males can be released manually or with uncrewed aerial vehicles (16). Although the complete elimination of females is not essential for SIT, it is believed to increase the efficacy of this control mechanism (17) and could decrease the cost of rearing both males and females. The release of males, which do not bite, is also generally more acceptable to people living in release areas and potentially to regulatory bodies (18).

Likewise, the incompatible insect technique (IIT) involves the release of male mosquitoes, males infected with a naturally occurring insect bacteria, *Wolbachia* (7, 8). The *Wolbachia*-infected males are released en masse to mate with wild type non-infected females, resulting in a lack of viable offspring due to cytoplasmic incompatibility (19). The success of IIT is dependent on limiting female contamination in the male releases; if infected females are released, this could lead to *Wolbachia* infections in natural mosquito populations and may reduce the effectiveness of future suppressive releases (20). In addition to SIT and IIT, a variety of genetic control technologies have emerged. These methods involve the release of non-biting male insects that have been genetically engineered to suppress mosquito populations or to render the mosquitoes resistant to human pathogens (12, 21, 22). The identification of scalable male mosquito sex sorting technology is therefore of critical importance to the further development, evaluation, and use of these emerging mosquito population control methods.

## 2 Criteria that define an ideal sorting mechanism

Manual sorting, including the use of sieving or glass plates to separate female pupae, which are larger in size, or the visual sorting of adults, can be sufficient at small scale (23, 24). However, these methods are not adequate for global deployment of emerging mosquito control technologies (25, 26). What criteria are essential for optimized scaled male mosquito production? Papathanos et al. (26) described several such criteria, which are summarized here (**Table 1**) and then discussed in relation to a variety of sorting technologies. Sorting methods should be stringent, scalable, cost-effective, efficient, and readily deployed at factory level. For cost-effectiveness, the method should ideally result in the early removal of females during development to decrease rearing costs. The sorting technology is ideally conditional, meaning that no permanent genetic modification of the mosquito strain is necessary. This is because it may be tedious, challenging, or impossible to introduce sorting mutations into the existing genetic background of the males to be released. Moreover, if regulatory permits for release of mosquitoes have already been procured, amendments to these permits could be required. Likewise, the sorting technology itself must also meet the regulatory standards of each country in which it will be implemented (25, 26) and is ideally acceptable to local stakeholders. The method should also be deployable globally, either through establishment of the sorting technique onsite or through the delivery of sorted mosquitoes, potentially to distant, remote, or resource-poor locations. The mechanism should be stable; for example, the rearing facilities should not need to consistently re-purify genetic strains, which should not be cumbersome to maintain. The technology should ideally be readily applicable to multiple disease vector mosquito species. Finally, it is essential that the sorted males are mating-competitive with respect to males present in nature (25, 26). Below, genetic sorting, automated sorting, and RNA interference (RNAi) yeast are evaluated using these criteria (**Table 1**).

**Table 1:** Comparison of manual, genetic, automated, and RNAi yeast-mediated male mosquito sorting technologies with respect to criteria that describe an ideal sorting technology.

Criterion	Mechanical	Genetic	Automated sorter	RNAi yeast
Simple	–	+	+	+
Stringent	–	+	+	–
Efficient	–	Variable; dependent on time required to maintain the strain	+	+
Not labor-intensive	–	Variable	+	+ (assuming yeast can be purchased)
Early removal of females	+	+	+	+
Conditional	+	Variable	+	+
Useful in multiple mosquito species	Variable	+ (but each strain must be constructed)	+ (but must be adjusted for each species)	+ (but yeast strains must be generated for additional species)
No impact on male mating-competitiveness	+	Has been achieved	Has been achieved	+ (based on lab studies; yet to be evaluated in the field)
Deployable in remote areas	+	Strain-dependent	+	+
Scalable	–	+	+	+
Meets regulatory standards	+	Must be approved by regulatory bodies in which it is used	+	Likely; but yeast import may need approval
Would not require modification of existing protocols	+	Variable	+	+
Cost-effective	–	Dependent on needs of maintaining the strain	+	TBD

Plus signs signify yes, while negative signs indicate no for each respective criterion.

## 3 Existing methods for male mosquito sex-separation

The generation of genetic sex sorting strains has been hotly pursued. These initially centered on methods of killing female mosquitoes while preserving male survival at a chosen time. Genetic sex sorting technologies were established in *Anopheles albimanus* by linking the propoxur resistance allele to the Y chromosome (27). Dieldrin resistance was linked to the Y chromosome to permit sex sorting of semi-sterile *Anopheles arabiensis* for SIT applications (28). In a modern version of similar technology, a conditional, self-limiting trait based on *doublesex* was linked to the tetracycline-off genetic switch to conditionally kill *Aedes aegypti* females (14). Eye color sexing strains were generated for *A. aegypti* (29), and a sex distortion transgene that selectively cleaves the X chromosome was introduced to *Anopheles gambiae* (30). Fluorescent markers were also introduced to facilitate machine-based sorting in several species (31, 32) including an *Anopheles stephensi* strain separable in the first instar (33). Efficient sex separation was achieved by exploiting differential alternative splicing of a dominant marker in *Aedes aegypti* (34). The precision-guided sterile insect technique (pgSIT) was used in *Aedes aegypti* to generate flightless females and sterile males that are deployable at any life stage, including through the release of eggs (35). Precision-guided sterile males were also generated using a binary CRISPR strategy to disrupt both female survival and male fertility in *A. gambiae* (22). Though powerful, pgSIT does still require the separation of males and females to establish the crosses that generate the offspring for releases. Additionally, the discovery that expression of the *Nix* male-determining transgene converts *Aedes albopictus* females to males (36) may assist with SIT technologies and could double male production. Thus many elegant genetic sorting strains have been designed for a variety of mosquito species.

Although a variety of cutting-edge genetic sorting methods have been developed, each sorting method must be generated separately in each species (**Table 1**). Moreover, whatever the genetic method utilized, it is critical that the resulting males are mating-competitive (37). Lab-reared genetically engineered *A. aegypti* were shown to compete effectively with wild males with respect to mating (38). The mating competitiveness of an *A. arabiensis* genetic sex sorting strain was demonstrated (39), and a GFP-sorting strain was also shown to be fit (32). *Aedes albopictus* females expressing *Nix* are also mating competitive (36). These studies collectively demonstrated that genetically engineered mosquitoes can effectively compete in the field (**Table 1**). However, while researchers generally agree that genetic sex sorting strategies are feasible, further genetic modification of existing strains could complicate production strategies or require the amendment of regulatory permits that might have already been attained. Additionally, public opposition to the release of transgenic mosquitoes still persists in critical locations (40, 41) and is an important criterion to consider and address (**Table 1**). To this end, Lutrat et al. (42) combined two sexing strains to enable the scaled production of non-transgenic *Aedes* spp. males, and genetic sex separation that enables production of non-transgenic *A. aegypti* males was recently reported (43). These genetic strategies are highly effective and will be useful assuming the mosquitoes can be produced on site or transported to remote locations (**Table 1**).

The need for sorting also resulted in the development of several automated sex-sorting tools. Crawford et al. (3) reported use of the Verily system for producing *Wolbachia-*infected *A. aegypti* mosquitoes for the *A. aegypti*-suppressing Debug program. It involves an automated larval rearing system that uses first instar larvae as an input for production of pupae. A multi-step sex separation method assesses sex-specific morphological differences following sieving, which is paired with custom industrial vision software that images males (3). The system boasts an estimated 1 in 26 billion female contamination rate. Although powerful, it was initially unclear if the technology could impact remote and resource-poor global locations. A study in Puerto Rico (44) suggested that packing and shipping sorted males had a small though significant impact on male fitness as measured by a mark-release-recapture strategy that detected significant daily male losses. These findings suggested that shipping and packaging practices needed to be improved. A field evaluation on the British Virgin Islands compared *A. aegypti* males produced and shipped 5,794 km vs. males produced on site. The improved shipping methods implemented in the study led to no detected impact on mosquito performance (45). An Australian team collaborated with DeBug to drive strong suppression of *A. aegypti* with a 0% female field contamination rate (7). These results indicate that the DeBug strategy, which reduces mosquito populations (3, 7, 46, 47), can have a global impact, especially if the cost is acceptable and the results can be further replicated in additional geographical settings and additional species (**Table 1**).

Gong et al. (40) reported the development of an automated pupal sex sorter which generated efficient sorting of *A. aegypti, A. albopictus*, and *Culex quinquefasciatus* at scale. The sorter, which combines a pupal source tank, a sorting glass for sex separation, and collection baskets for male and female larvae, enabled 99.5% successful separation of males, which had comparable flight abilities to mosquitoes sorted manually. Production of males was 17x higher than manual sorting with a Fay-Morlan (23) glass sorter. The performance of released incompatible males was evaluated at an experimental field site and compared to natural males at a control site. Mating performance was estimated to be higher for *A. albopictus* incompatible males. Significant declines in the number of female adults and hatched larvae were observed at the release site. The field trials were performed in *A. albopictus* mosquitoes, and the sorter is reported to be useful in *A. aegypti* and *C. quinquefasciatus*, which can be assessed in future trials. The authors noted that the system is currently being used in trials conducted in Austria, Brazil, China, Italy, and the United States, suggesting that it is globally deployable.

Mamai et al. (48) recently compared the Orinno and WOLBAKI automated sex sorters. The ORINNO sorter (48) separates pupae on the basis of sexual dimorphism and is being used to separate *A. aegypti* in a Singapore SIT/IIT program. The WOLBAKI sex sorter, based on the Fay-Morlan manual sorter (23), was lab-validated in both *Aedes* spp. and *Culex quinquefasciatus* (40) and during an *A. albopictus* IIT program in China (6). Both systems sorted *A. aegypti* with less than 1% female contamination, low pupal mortality rates, and high flight capacities in sorted males. The WOLBAKI sorter also worked well for *A. albopictus*, for which the ORINNO sorter had lower efficacy, with female contamination rates >1% and pupal mortality. The study highlighted the importance of evaluating different species of mosquitoes and considering the specific needs of the mosquito control program in which a sorter is implemented.

## 4 RNAi-mediated sex-sorting applications

**Figure 1:**
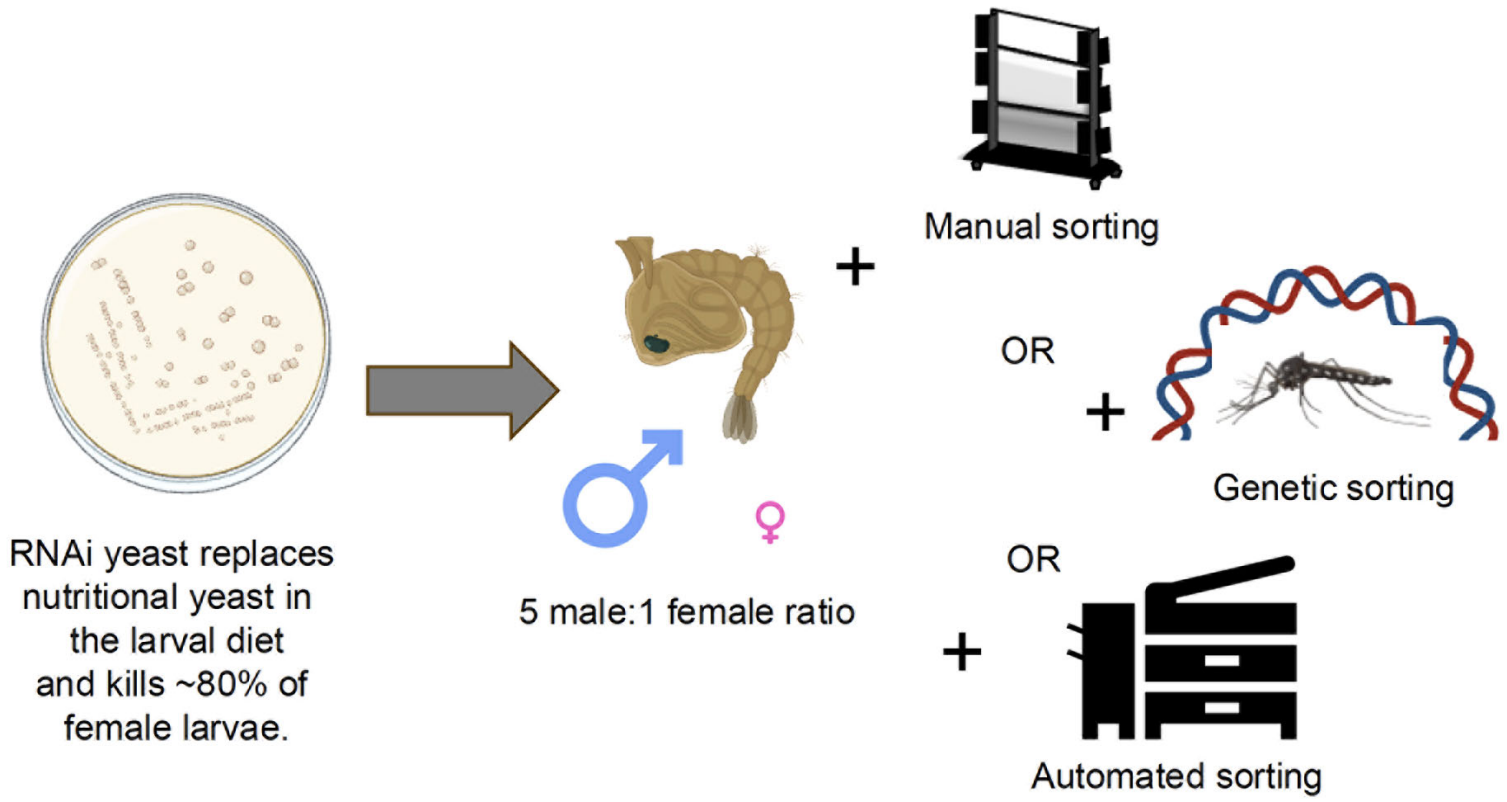
Potential RNAi yeast, manual sorting, genetic sorting, and automated sorting combinations for male mosquito production. A variety of sex sorting methods for male mosquito sorting have been developed. The use of RNAi yeast during mosquito rearing could support manual, genetic, and automated sorting technologies, potentially enabling more cost-effective, efficient, and effective sex-sorting.

RNAi, which provides a means of silencing genes, is a natural cellular process that has been exploited in the laboratory to discover female-specific lethal genes that selectively kill females when silenced (49–52). To develop scalable RNAi yeast sex-separation technology, short hairpin RNA (shRNA) was expressed in *Saccharomyces cerevisiae* (baker’s yeast) and subsequently fed to larvae. *Pichia pastoris* (53), *Escherichia coli* (49), and *S. cerevisiae* (54) have been successfully used for oral RNAi in *A. aegypti. S. cerevisiae* is a model organism that is genetically tractable, inexpensive to culture at scale, and which enables cost-effective shRNA production during yeast propagation (**Table 1**) (55). Moreover, the yeast is highly attractive to mosquito larvae, which readily consume it, resulting in targeted gene silencing (54). Given these attributes, as well as the high level of enthusiasm among stakeholders for the use of baker’s yeast to target mosquitoes (56), *S. cerevisiae* was chosen for the development of a scalable microbial interfering RNA production and delivery system. A yeast RNAi screen in *A. aegypti* (50) uncovered many previously uncharacterized female-specific larval lethal genes, including the *gamma-glutamyl transpeptidase (GGT)* gene, which is conserved among mosquitoes (52). Yeast consumption resulted in ~80% female lethality (**Figure 1**, **Table 1**), yet did not impact males, which were fit and five times more abundant than females. The results in *A. aegypti* experiments were applied to other mosquito species (**Table 1**). Silencing the *A. albopictus*, *A. gambiae, C. quinquefasciatus* and *Culex pipiens GGT* genes yielded comparable results. Moreover, the yeast could be heat-inactivated, dried, and incorporated into normal larval mass rearing diets, permitting scaled production of fit adult male mosquitoes of each species (**Table 1**).

The yeast strains used in these initial proof of concept studies (50–52) are not suitable for industrial-scale yeast production. To address this, Super PiggyBac/Cas-CLOVER was used to generate yeast strains containing multiple copies of *GGT* shRNA expression cassettes that had been stably integrated into the yeast genome, and in which genetic auxotrophies had been restored (57). The yeast, in which ~30x higher shRNA expression was detected, was used for scaled rearing of *C. quinquefasciatus* and *C. pipiens*, in which smaller amounts of yeast were required for scaled adult male production. Similar yeast strains can be generated for additional mosquito species (**Table 1**). Kilogram-scale yields of the commercial-ready *Culex* male separation yeast were achieved in pilot large-scale fermentations, indicating that the yeast is suitable for industrial scale production. These scaled fermentations demonstrated that production of the yeast required no specialized media, indicating that the yeast can be cheaply produced in production schemes which mimic those of typical commercial nutritional yeast fermentations. Thus, it is anticipated that once the yeast is produced, it can be heat-inactivated, dried and shipped to mosquito mass-rearing facilities, where it can be used in place of nutritional yeast, a common component of mass-rearing diets, at potentially no extra cost to the rearing facilities. Facilities that wish to adopt the scaled rearing protocols used by Brizzee et al. (57) could expect to generate an estimated 500,000 male mosquitoes per kg of heat-inactivated dried RNAi yeast, which costs ~$1.50 USD/kg based on current wholesale production costs for bulk animal feed-grade dried inactivated nutritional yeast in the United States (58).

It should be noted that the RNAi yeast technology utilizes a genetically modified organism, but it is a dead microbial to be used only within the rearing facility. It is anticipated that indoor use of female-specific yeast-based RNAi larvicides (52) may inexpensively facilitate population-based control strategies in a manner that would be acceptable to regulatory agencies. With an 80% kill rate, the technique is not a stand-alone separation method but could be combined with other sorting technologies (**Figure 1**, see below). Moreover, if RNAi yeast use is to be paired with sterilizing irradiation, both the males and contaminating females would likely be sterile, making the female releases less problematic. Given that yeast is inexpensive to produce and easy to ship worldwide, the technology could potentially be impactful at a global scale (55) (**Table 1**).

## 5 Discussion

This review has highlighted the need for effective male sex sorting technology, discussed criteria that define an ideal technology, and evaluated if genetic, automated, and RNAi sorting technologies match these criteria (**Table 1**). Various genetic technologies, which differ for each mosquito species, have differing capabilities of meeting these needs. However, the efficacy of genetic systems can be extremely high, off-setting the time and associated costs that could be required for strain maintenance, which is being addressed with new strains that are self-sustaining (14, 43). Automated sorters can clearly be highly impactful, particularly if the mosquitoes sorted (45) or the sorters themselves (40) can be shipped and used globally at a reasonable price. Finally, RNAi yeast meets many of the criteria noted in **Table 1** with one critical exception: ~20% of females survive. However, assuming that the yeast is available at little or no extra cost to the users who already use nutritional yeast in their rearing diets, it could be paired with some genetic, automated, or even manual sorting programs, potentially reducing female contamination rates and overall costs while allowing more efficient male generation (**Figure 1**). For example, as recently observed following 15% sodium chloride male-sorting pre-treatments of *A. aegypti* larval-pupal mixtures prior to manual or automated sorting (59, 60), RNAi yeast treatments of larvae could streamline sorting, increase load capacities, and reduce manual operations in a threshold-dependent manner that is more exaggerated when the number of males exceeds 180,000 for manual sorting and 200,000 for automated sorting. Combining RNAi yeast with manual sorting may be sufficient for regional control districts to deploy SIT or IIT.

Of course, for technologies such as SEPARATOR (33, 34) that sort in the first instar or earlier and/or in which female pupal recovery is already low after using a single method of sex separation, the use of yeast may not significantly increase male purity levels or efficiency and may not be warranted. In these instances, RNAi yeast could still potentially assist with the sorting of males used to produce genetic strains. Nevertheless, as recommended for those considering the integration of sodium chloride treatments into mass-rearing protocols, the use of RNAi yeast should be guided by thoughtful consideration of rearing efficiency and operational scale (60). Furthermore, moving forward, when sex separation techniques and population control strategies are combined, in addition to measuring entomological endpoints, it will be critical to consider if disease incidences are reduced. Although epidemiological endpoints can be challenging to assess (61), population control technologies can have a significant impact on disease incidence (46, 47) when the sex-sorting barrier is eliminated.

Another area of interest is the extension of sex-separation methods to other insect pests of agricultural, veterinary, human disease, or nuisance-related interest. The International Atomic Energy Agency (IAEA) has led efforts to explore and optimize genetic, molecular, behavioral, and mechanical sex separation in mosquitoes (48, 60, 62–65). Their research interests span across many insects (66), including tsetse flies (67, 68), moths (69, 70), and various fruit fly pests (71, 72), for which they are working to address gaps associated with mass rearing, genetics, microbiology, sterilization, behavior, transport, release, and quality control. Yeast is readily eaten by a number of these insects, opening the potential for RNAi-based male production and sex separation. This is particularly of interest for insects in which transgenic technologies have yet to be established. For insects with sequenced genomes, the straightforward production of RNAi yeast targeting female-specific lethal genes (57, 73) could enable sex separation that may otherwise be challenging to achieve. The development of RNAi-mediated, mechanical, genetic, or automated sorting technologies, and combinations of these methods, will facilitate the implementation of population control methods for a wide variety of insect pests.
